# A targeted boost-and-sort immunization strategy using *Escherichia coli* BamA identifies rare growth inhibitory antibodies

**DOI:** 10.1038/s41598-018-25609-z

**Published:** 2018-05-08

**Authors:** Rajesh Vij, Zhonghua Lin, Nancy Chiang, Jean-Michel Vernes, Kelly M. Storek, Summer Park, Joyce Chan, Y. Gloria Meng, Laetitia Comps-Agrar, Peng Luan, Sophia Lee, Kellen Schneider, Jack Bevers, Inna Zilberleyb, Christine Tam, Christopher M. Koth, Min Xu, Avinash Gill, Marcy R. Auerbach, Peter A. Smith, Steven T. Rutherford, Gerald Nakamura, Dhaya Seshasayee, Jian Payandeh, James T. Koerber

**Affiliations:** 1 Department of Antibody Engineering, Genentech, 1 DNA Way, South San Francisco, California, 94080 USA; 2 Department of Infectious Diseases, Genentech, 1 DNA Way, South San Francisco, California, 94080 USA; 3 Department of Structural Biology, Genentech, 1 DNA Way, South San Francisco, California, 94080 USA; 4 Department of Biochemical and Cellular Pharmacology, Genentech, 1 DNA Way, South San Francisco, California, 94080 USA; 5 Department of Translational Immunology, Genentech, 1 DNA Way, South San Francisco, California, 94080 USA; 6 Department of BioMolecular Resources, Genentech, 1 DNA Way, South San Francisco, California, 94080 USA

## Abstract

Outer membrane proteins (OMPs) in Gram-negative bacteria are essential for a number of cellular functions including nutrient transport and drug efflux. *Escherichia coli* BamA is an essential component of the OMP β-barrel assembly machinery and a potential novel antibacterial target that has been proposed to undergo large (~15 Å) conformational changes. Here, we explored methods to isolate anti-BamA monoclonal antibodies (mAbs) that might alter the function of this OMP and ultimately lead to bacterial growth inhibition. We first optimized traditional immunization approaches but failed to identify mAbs that altered cell growth after screening >3000 hybridomas. We then developed a “targeted boost-and-sort” strategy that combines bacterial cell immunizations, purified BamA protein boosts, and single hybridoma cell sorting using amphipol-reconstituted BamA antigen. This unique workflow improves the discovery efficiency of FACS + mAbs by >600-fold and enabled the identification of rare anti-BamA mAbs with bacterial growth inhibitory activity in the presence of a truncated lipopolysaccharide layer. These mAbs represent novel tools for dissecting the BamA-mediated mechanism of β-barrel folding and our workflow establishes a new template for the efficient discovery of novel mAbs against other highly dynamic membrane proteins.

## Introduction

The emergence of multi-drug resistant bacteria is a global health crisis that demands the discovery of new antibiotics, which requires a better understanding of microbial physiology and vulnerabilities. Integral outer membrane proteins (OMPs) reside within the distinctive outer membrane (OM) of Gram-negative bacteria where they perform diverse functions critical for bacterial survival^1,2^. OMP functions include roles in active transport and diffusion of nutrients, efflux of toxic molecules, and construction of the OM permeability barrier. OMPs assume a common fold known as the β-barrel and are inserted into the OM by the essential β-barrel assembly machinery (BAM) complex^3,4^, of which BamA is itself an OMP and a central component of the BAM complex. For the BAM complex to accomplish its essential OMP-foldase function, it has been postulated to undergo rapid and extensive conformational changes. Recent x-ray crystal and electron microscopy structural studies have suggested that the BamA β-barrel can assume inward- and outward-facing states that differ by a 65° rotation and 15 Å shift within the extracellular exit pore region^5–7^. In fact, the extent of conformational change proposed for BamA is among the largest described for any previously reported membrane protein structure to date. Obtaining a detailed mechanistic understanding of essential OMPs like BamA may ultimately enable the design of future therapeutics targeting Gram-negative bacteria.

To date, genetic tools have greatly facilitated mechanistic studies of BamA, but gene knockouts or mutations do not necessarily mimic the effects produced by potential small molecule or protein-based inhibitors. The discovery of a peptidomimetic that interacts with many OMPs, including BamA, and possesses antimicrobial activity further highlights the potential of OMP-targeted therapeutics^8^. However, potent and highly selective pharmacological tools to modulate the function of BamA have not yet been reported. Here, we hypothesized that antibody-mediated interference through an extracellular epitope of BamA could inhibit the essential OMP-folding function, resulting in bacterial growth inhibition and cell death. To our knowledge, this proposed mechanism of direct bacterial growth inhibition by a naked anti-OMP mAb has not been previously demonstrated on any pathogenic or laboratory strain^9^.

The use of monoclonal antibodies (mAbs) have revolutionized the study of mammalian membrane protein function through Western- and FACS-based expression profiling, as chaperones for high-resolution structural studies, and, in rare cases, as proof-of-concept agonists or antagonists^10–12^. For bacterial OMPs, while mAbs useful for Western or FACS analysis can often readily be obtained, remarkably few agonists or antagonists have been described, potentially due to masking of functional epitopes by lipopolysaccharide (LPS)^13,14^. Limitations in commonly used mAb discovery methods can also partially account for this gap. First, antibody phage display selections against bacterial OMPs can be challenging due to the high homology between the target OMP and host proteins in the strain used to amplify the libraries^15^. Second, immunization-based methods are resource intensive and inefficient, and replete with uncertainties related to antigen format (summarized in Supplementary Table 1). While highly desirable, the production of sufficient purified full-length membrane protein (MP) antigens stabilized in various matrices (*e*.*g*., detergent, liposomes, nanodiscs, amphipols or styrene-maleic acid polymers) remains challenging, resource-intensive, and may not guarantee targeting of the native form of the MP, particularly if mutagenesis is required for recombinant expression or stable purification^12,16–19^. Finally, native antigen formats, such as membrane fractions, membrane vesicles, or whole cells, often give rise to significant titers against non-target proteins, thus leading to high backgrounds and especially low mAb discovery efficiencies.

Adding to the aforementioned complexities, the distinct structural and functional characteristics of OMPs like BamA may further confound mAb discovery efforts. Specifically, the extracellular epitopes on many OMPs are inherently dynamic, may change conformation depending on the lipid or host environment, and may be masked by LPS^20,21^. Additionally, mAbs capable of functionally modulating an OMP into an activated or inhibited state have not been reported to date, and are therefore likely to be rare. Faced with these challenges, we endeavored to streamline anti-BamA mAb discovery by optimizing antigen formats (reconstitution matrices and native whole cells), adjuvants, immunization strategy, BamA-specific cell sorting, and high-throughput hybridoma purification. Notably, while our extensive traditional mAb discovery efforts failed to identify any functional anti-BamA mAbs, our optimized workflow improved the efficiency of FACS^+^ anti-BamA mAb generation by >600-fold and enabled the discovery of rare anti-BamA mAbs with direct growth inhibitory activity in the presence of a truncated LPS layer. These growth inhibitory anti-BamA mAbs will enable deeper mechanistic studies of BamA function and further interrogation of BamA as a novel therapeutic target.

## Results

### Evaluation of adjuvants and matrices with purified MP antigen

**Figure 1:**
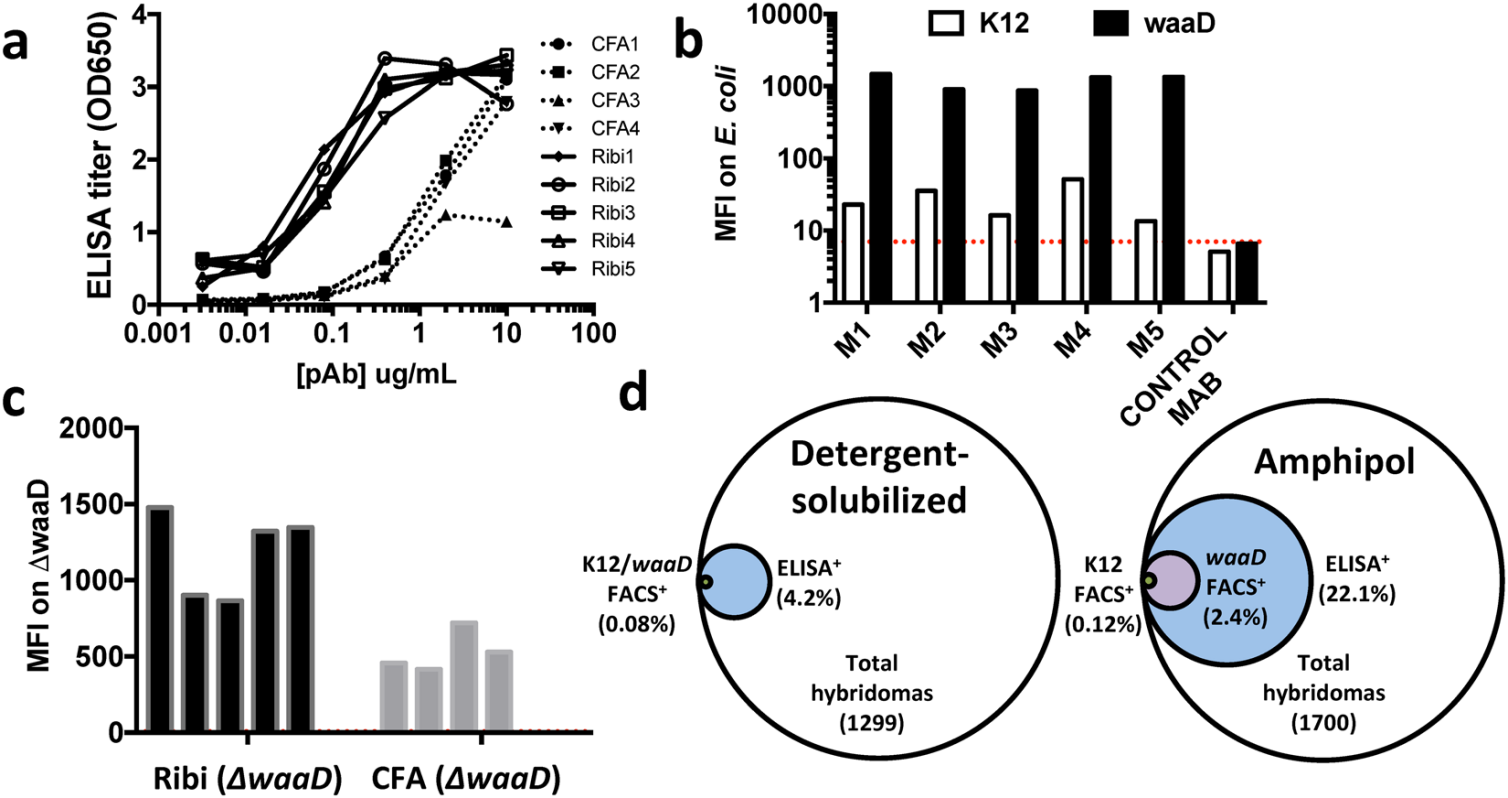
Immunization with amphipol-BamA and Ribi adjuvant generates robust antibody response. (**a**) Anti-BamA pAb titers using purified pAbs were measured by ELISA for BamA immunized mice with either Ribi (n = 5; solid lines) or CFA (n = 4; dashed lines) adjuvants. (**b**) Mean fluorescent intensity (MFI) as determined by flow cytometry of 40 μg/mL BamA pAbs against Δ*waaD* (black box) or K12 strains (white box). M1-M5 represent individual mice from the Ribi immunized group. MFI for irrelevant antibody control is depicted by red dashed line. (**c**) MFI values for individual mice from either the Ribi adjuvant group (black box) or CFA adjuvant group (grey box). (**d**) Summary of ELISA and FACS results for panel of hybridomas from mice immunized with detergent-solubilized or amphipol-BamA.

After we established methods to produce high-quality recombinant *E*. *coli* BamA reconstituted into a non-detergent amphipol matrix (see Methods), we set out to evaluate the impact of two commonly used adjuvants on the success of mAb discovery against this dynamic and essential OMP. Balb/c mice were immunized with BamA-amphipol using either CFA (Complete Freund’s Adjuvant) or Ribi adjuvants. All mice showed a strong immune response against BamA as measured by ELISA with the CFA adjuvant yielding antibody titers ~10× lower compared to the Ribi adjuvant (Fig. 1a). To monitor binding to extracellular epitopes of BamA, we used a FACS-based *E*. *coli* whole cell binding assay. However, because Gram-negative bacteria like *E*. *coli* possess LPS in their outer membrane which hinders access of antibodies to bacterial OMPs^22^, we screened the polyclonal antibodies (pAbs) by FACS on K-12 *E*. *coli* and a Δ*waaD E*. *coli* mutant strain, which possesses the most truncated form of LPS that still supports bacterial growth (Supplementary Fig. 1)^22–24^. As predicted, we observed increased FACS signal with the Δ*waaD* strain compared to K-12 *E*. *coli*, confirming that elaborated LPS can prevent binding of most antibodies to OMPs like BamA (data from Ribi immunized mice are shown in Fig. 1b). Nevertheless, all pAbs showed some FACS binding to the K-12 strain, suggesting that certain mAbs are able to bind BamA in the presence of the K-12 core-LPS (Fig. 1b). Notably, immunizations of the BamA-amphipol complex with Ribi adjuvant yielded higher FACS signals compared to those with the CFA adjuvant (Fig. 1c), consistent with the observed ELISA results (Fig. 1a). Therefore, Ribi was the adjuvant selected for all subsequent immunizations.

In favorable cases, recombinant purified MPs can be prepared in a range of solubilizing matrices. We next tested the effect of select protein matrices on the anti-BamA antibody response, in part, motivated by the hypothesis that different matrices may present more membrane proximal epitopes. Using Ribi adjuvant, we immunized mice with amphipol-reconstituted BamA or a short-chain detergent-solubilized BamA antigen and generated hybridomas from both groups. Because we observed that immunizations with amphipol-reconstituted BamA generated some non-specific antibodies against the amphipol matrix, we performed all ELISAs with detergent-solubilized BamA in order to decrease non-specific binding. In the short-chain octyl-glucoside detergent-solubilized format, intended to promote BamA conformational dynamics and maximum exposure of extracellular epitopes, only 54 out of 1299 IgG^+^ supernatants (4.2%) bound to BamA by ELISA. In the amphipol format, chosen because of its higher recovery yields compared to liposome incorporation, 375 out of 1700 IgG^+^ supernatants (22.1%) bound to BamA by ELISA (Fig. 1d). Intuitively, the 5-fold increase in antigen^+^ (Ag^+^) hybridomas could be due to greater *in vivo* stability of the amphipol format over the detergent-solubilized material^25^. Subsequent FACS screening of the panel from the BamA-amphiphol immunized group identified that ~2.4% of the IgG^+^ supernatants were FACS^+^ on the Δ*waaD* strain, including several clones that also bound to the K-12 strain (Fig. 1d). As we ultimately sought to identify potentially growth inhibitory mAbs, we tested the ability of purified FACS^+^ mAbs to inhibit growth of the Δ*waaD* mutant without any additional immune system components such as complement. However, despite robust immune responses that led to the discovery of many FACS^+^ anti-BamA mAbs, none of these mAbs were biologically active.

### Combining native and recombinant antigen formats

**Figure 2:**
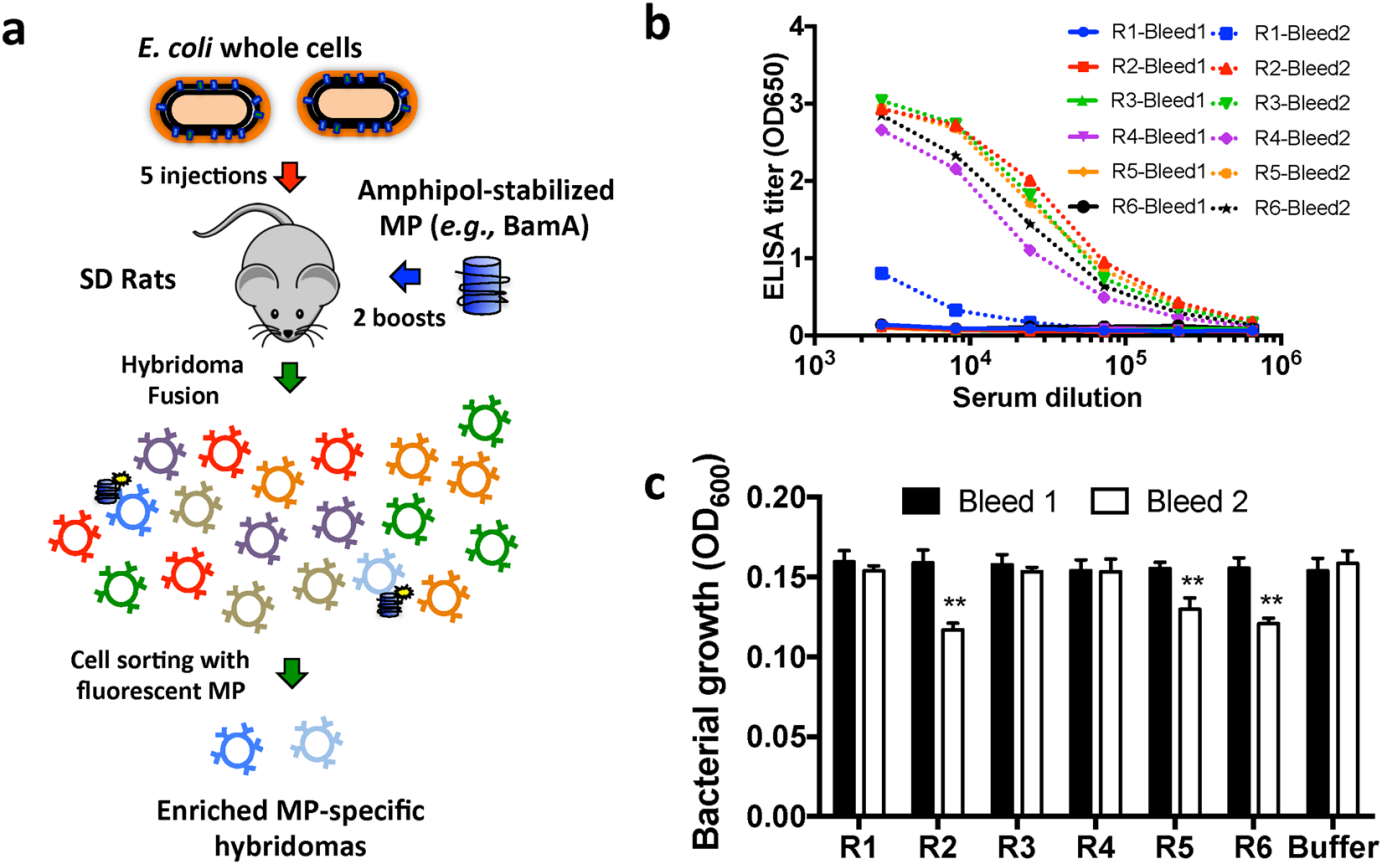
Combining native and recombinant BamA formats enables discovery of growth inhibitory pAbs. (**a**) Schematic of “targeted boost-and-sort” strategy in which Sprague-Dawley (SD) rats are initially primed with K12 bacteria (red arrow) followed by two boosts with the recombinant amphipol-BamA (blue arrow). After subsequent cell fusion to generate hybridomas, fluorescently-labeled BamA is used to single cell sort BamA^+^ hybridomas to generate a final enriched panel. (**b**) pAb ELISA titers using purified pAbs dramatically improve post-protein boost (dashed line) compared to pre-boost (solid line). (**c**) pAbs from three rats (R2, R5, and R6) reduce growth of Δ*waaD E*. *coli* as measured by OD600 density only after protein boost (white vs. black box). Growth differences observed for bleed 2 in R2, R5, and R6 were statistically significant compared to buffer controls (n = 3, p < 0.01).

We hypothesized that our failure to obtain inhibitory anti-BamA mAbs might be due to a limited sampling of the BamA extracellular epitopes or poor targeting of the OM-relevant conformations of BamA. Indeed, recent high-resolution structural studies have posited that BamA is a highly dynamic MP that likely exists in multiple conformations relevant for its function^3,5,6^. To explore a larger range of potentially relevant BamA conformations during antibody generation, and also provide a more relevant OM context for presentation, we revised our immunization strategy to employ whole bacterial cells (Fig. 2a). In this respect, it is particularly important to note that BamA is estimated to be present at only ~1500–4000 copies per cell, and therefore represents a minor fraction of the total cellular protein in *E*. *coli*^26,27^. Accordingly, we also switched from mice to rat immunizations because rats can tolerate larger doses of whole bacteria and a higher number of B cells can be recovered post-immunization. We reasoned that higher doses of bacteria and more recovered B cells should enhance our initial sample size to aid in the recovery of potentially rare functional anti-BamA mAbs.

Following the above rationale, after five sub-lethal injections of live K-12 *E*. *coli*, all immunized animals failed to show significant pAb binding to BamA by ELISA (solid lines in Fig. 2b). However, an anti-BamA response against purified BamA could be detected by Western blot, suggesting the presence of some anti-BamA titers and antibodies (Supplementary Fig. 2). To boost the recovery of BamA-specific antibody responses, we next challenged these same rats with amphipol-reconstituted purified BamA, but without the use of adjuvant in order to avoid initiating a new immune response. After the two injections of only 10 μg of purified protein antigen per rat, we observed a dramatic increase in the anti-BamA titers (dashed lines in Fig. 2b). We assayed the pAbs by FACS, but as expected, were unable to verify a BamA-specific FACS response due to the high background of antibodies against other components of the bacteria (Supplementary Fig. 3). Nevertheless, we wanted to determine whether this new hybrid immunization strategy that produced high anti-BamA-specific titers led to mAbs with distinct biological activity. We therefore tested the ability of pAbs from before and after the BamA protein boost to inhibit bacterial cell growth. Remarkably, pAbs from several rats slightly reduced bacterial growth only after the BamA-amphipol protein boost (black vs. white bars in Fig. 2c, p < 0.01). Because this weak growth effect was only observed after boosting with amphipol-reconstituted BamA, we predicted that the inhibitory effect was likely due to a specific anti-BamA response and that only a small fraction of the anti-BamA mAb pool was functional.

### MP-specific sorting and high-throughput production of hybridomas

**Figure 3:**
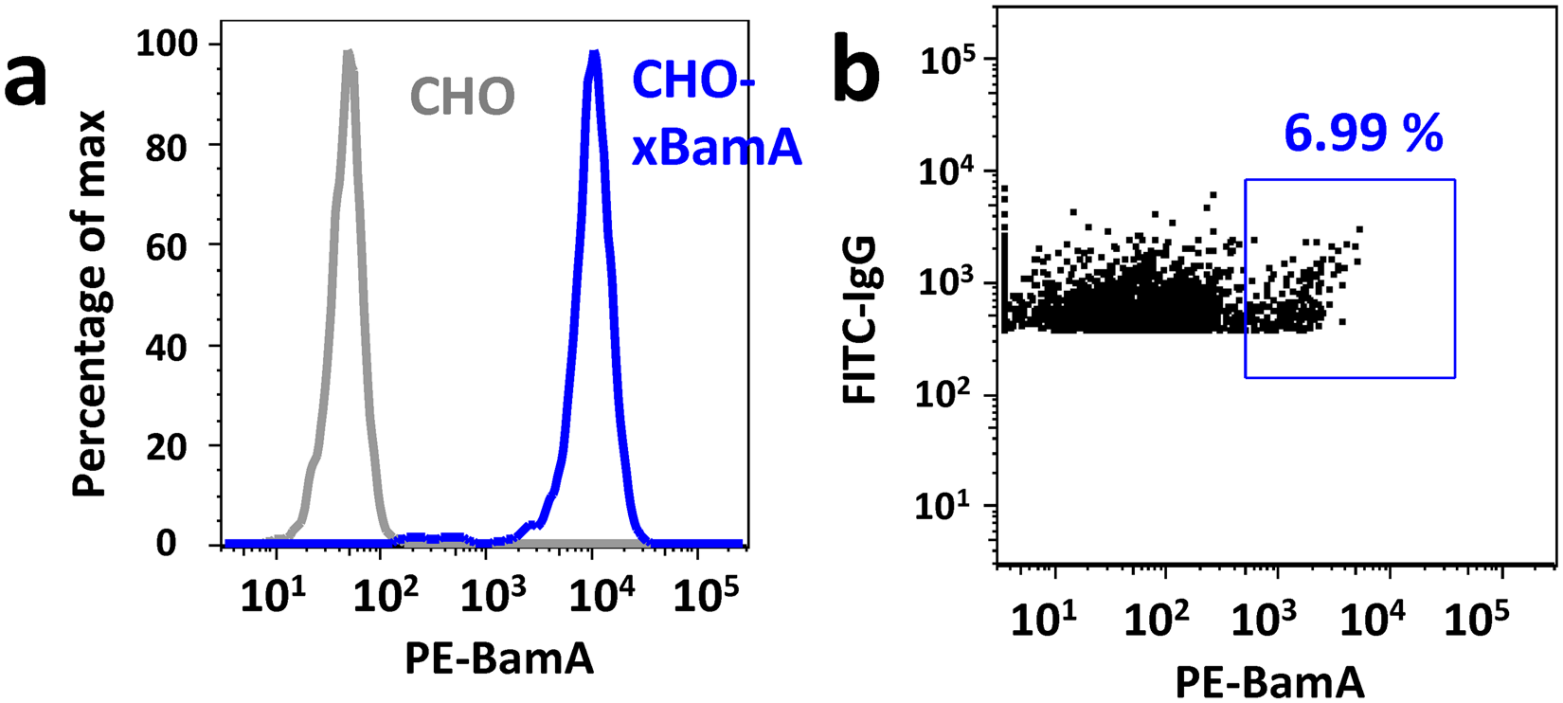
Amphipol-reconstituted BamA enables sorting of BamA^+^ mock B cells and hybridomas. (**a**) PE-labeled BamA only stains CHO cells transiently expressing a GPI-anchored anti-BamA mAb (blue) and not CHO cells without mAb (grey). (**b**) Representative sorting profile for BamA^+^ and IgG^+^ SP2ab rat hybridomas.

Since the percentage of IgG^+^ and Ag^+^ hybridomas are often extremely low, especially for complex and dynamic MP antigens like BamA, it is labor and resource intensive to generate a large panel of Ag^+^ clones for functional screening. Furthermore, traditional hybridomas do not display IgG on the cell surface, and hence, cannot be directly sorted for antigen-specific binding. To overcome these traditional bottlenecks to mAb discovery, we hypothesized that we could employ amphipol-reconstituted BamA to directly sort BamA^+^ hybridomas generated with a specialized myeloma fusion partner (SP2ab) that enables the production of both secreted and surface displayed IgG^28^. Hybridomas generate transcripts for both the secreted and membrane bound forms of IgG via alternative splicing. However, in the absence of co-expression of the Igα and Igβ (both components of the B-cell receptor complex) the membrane bound form of IgG is not stably displayed on the cell surface. The SP2ab fusion partner expresses both Igα and Igβ to enable surface display of IgG in addition to IgG secretion. To first verify that fluorescently labeled amphipol-reconstituted BamA could detect Ag^+^ cells, we generated a CHO cell line that transiently expressed a murine anti-BamA antibody on the surface via a glycosylphosphatidylinositol anchor^29^. Phycoerythrin (PE)-labeled BamA successfully stained the CHO cells with surface IgG, but not control CHO cells (Fig. 3a). Having validated that nanogram quantities of fluorescently labeled BamA should enable staining of BamA-specific cells, we generated hybridomas from the above immunized rats using the SP2ab fusion partner and sorted double positive (IgG^+^ and BamA^+^) hybridomas into individual wells of 96-well plates using a total of only 5 μg of PE-labeled amphipol-reconstituted BamA material (Fig. 3b).

**Figure 4:**
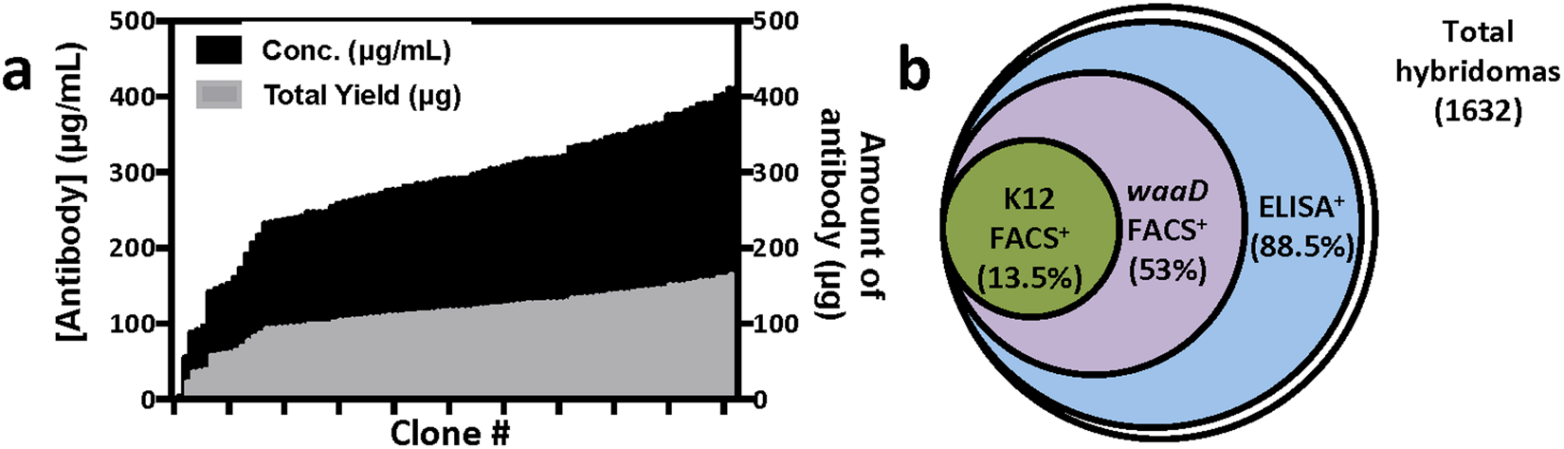
Sorting hybridomas with amphipol- BamA followed by high-throughput hybridoma production yields large panel of purified BamA^+^ mAbs. (**a**) Representative yields (~100 μg on average; grey bars) and final purified concentrations (~150 μg/mL on average; black bars) from one plate of purified hybridomas. (**b**) Panel of 1632 BamA-sorted purified hybridomas is highly enriched for BamA binding by ELISA (~88.5%), Δ*waaD* FACS (53%), or even K12 FACS (13.5%).

Most robust functional assays utilized in successful mAb discovery efforts, like our bacterial growth inhibition assay, require concentrated and purified mAbs for direct testing; and this represents a significant technical and practical bottleneck when there is a need to evaluate large mAb panels. To overcome this traditional screening impediment, we developed an efficient, high-throughput hybridoma culture and purification process. In brief, single BamA-sorted hybridoma cells were grown for one week in plates followed by another ten days in deep well blocks in a shaking incubator. Supernatants were then purified using an automated 1 mL Protein A purification process to yield final purified IgGs in a smaller volume (400 μL) for functional screening. The final purified mAb concentrations exceeded 100 μg/mL in ~95% of the samples with total yields of ~100 μg (Fig. 4a). We subsequently screened roughly one quarter of 1632 purified hybridomas by ELISA and FACS to quantify our antigen-specific sorting efficiency. Strikingly, 88.5% of all tested hybridomas were BamA ELISA^+^ and 53% were Δ*waaD* FACS^+^ (Fig. 4b). These 5 and 20-fold efficiency increases in identifying ELISA^+^ and FACS^+^ mAbs over our more traditional discovery methods described above (Fig. 1d) highlights the efficiency and robustness of our hybrid immunization targeted boost-and-sort platform to deliver a large panel of purified Ag^+^ clones for functional screening against a challenging MP target like BamA.

### Identification and characterization of growth inhibitory anti-BamA mAbs

To first characterize mAbs isolated from our workflow described above, epitope binning and affinity measurements were performed on a select panel of anti-BamA rat mAbs to characterize their binding properties (see below). For comparison, we also included several non-inhibitory mouse anti-BamA mAbs isolated from our traditional protein-only based hybridoma campaign (described above). Overall, the affinities of the mAbs ranged from 50 pM to 100 nM, with no clear correlation between affinity and discovery method (Table 1). In contrast to the high milligram amounts of purified BamA protein consumed by our traditional discovery efforts (Fig. 1d), this key result establishes that combining native and readily available BamA in the form of whole live bacteria with only a minimal amount of purified protein antigen for boosting and Ag-specific sorting (105 μg in total) can deliver a large panel of high-affinity and highly-selective mAbs (Fig. 4b).

**Table 1:** Summary of functional properties of anti-BamA mAbs.

% Growth inhibition	# of mAbs	KD range (nM)
<50%	1554	n.d.
50–75%	59	~0.05–100
76–90%	12	~0.1–20
>90%	7	~0.1–40

Affinity values for a subset of the anti-BamA mAbs as determined by Wasatch. All inhibitory mAbs bind by ELISA to only *E. coli* BamA and not *Entero* or *Kleb* BamA.

**Figure 5:**
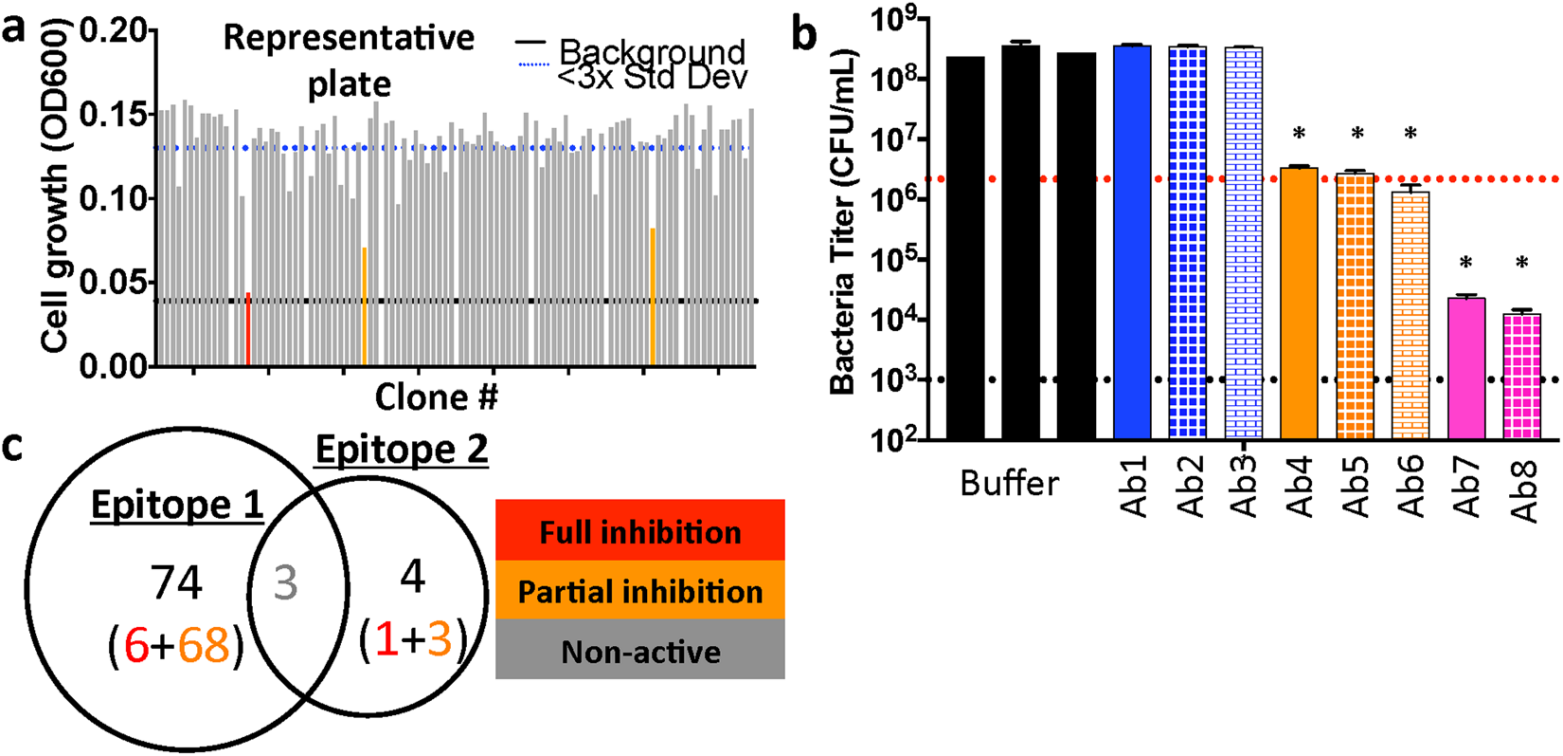
Functional and biochemical properties of rare panel of inhibitory anti-BamA mAbs. (**a**) OD600 values for Δ*waaD* strain after treatment with one representative plate of anti-BamA mAbs at 10 μg/mL for 4 hours. Analysis reveals the presence of rare partially inhibitory (orange lines) or fully inhibitory (red line) anti-BamA mAbs. Black line indicates lower limit of detection and dashed blue line indicates 3 standard deviations below buffer control. (**b**) CFUs were quantified for the Δ*waaD* strain at 0 hrs and 4 hrs after treatment with a subset of anti-BamA mAbs or buffer at 10 μg/mL (*p < 0.001 compared to buffer controls). Red dashed line represents CFUs measured at 0 hr and black dashed line represents lower limit of detection. Experiments were performed in biological triplicate. (**c**) Venn diagram of epitope-binning results on panel of anti-BamA mAbs reveals two epitopes that partially overlap. Bin 1 contains 6 full inhibitors (red) and 68 partial inhibitors (orange), whereas fewer mAbs (1 full and 3 partial inhibitors) occupy bin 2. Interestingly, non-inhibitory clones (grey) can block binding of mAbs in both epitope bins.

We next wished to determine if any of the mAbs we isolated inhibited the growth of *E*. *coli* considering that no anti-OMP antibodies with growth-inhibitory properties have been described to date. As such, all 1632 purified mAbs isolated from the targeted boost-and-sort approach described above were screened in a bacterial growth assay using both K-12 and Δ*waaD E*. *coli*. Consistent with the results from all of our previous anti-BamA discovery efforts, 95% of the screened rat mAbs had no (or minimal) impact on bacterial growth of the Δ*waaD* or K-12 *E*. *coli* strain. In contrast, and most remarkably, 5% of the rat mAbs partially or fully inhibited growth of the Δ*waaD E*. *coli* strain (see orange and red bars for a subset shown in Fig. 5a). In total, we identified 71 mAbs that partially inhibited growth (>50% growth inhibition) and 7 mAbs that fully inhibited growth (>90% growth inhibition) of the Δ*waaD E*. *coli* strain. To further characterize these mAbs, we measured the change in the number of viable bacteria after growth in the presence of a subset of these antibodies. We observed a ~50-fold increase in colony forming units (CFUs) when the Δ*waaD E*. *coli* strain was grown in the presence of buffer or non-inhibitory mAbs (black and blue bars in Fig. 5b). Bacterial growth in the presence of several partially inhibitory mAbs led to no change in CFU, suggesting these mAbs are bacteriostatic (orange bars in Fig. 5b, p < 0.001). Finally, and most remarkably, growth in the presence of the full inhibitors resulted in ~50–100-fold decrease in CFUs, suggesting that these mAbs are bactericidal (pink bars in Fig. 5b, p < 0.001). These data collectively suggest that we have identified naked anti-OMP mAbs capable of inhibiting growth of an *E*. *coli* Δ*waaD* strain in the absence of other immune effectors.

We found that the majority of the inhibitory anti-BamA mAbs occupied a single epitope bin on BamA (Fig. 5c). Incredibly, we note that several of the non-functional mouse mAbs also binned within this same epitope, suggesting that our targeted boost-and-sort discovery method is capable of more in-depth sampling of a given epitope than traditional mAb discovery efforts. To confirm that the unique inhibitory mAbs were specific to *E*. *coli* BamA, we tested and observed that there was no cross-reactivity by ELISA with the closely related BamA homologs from *Klebsiella pneumonia* and *Enterobacter cloacae* (Table 1). Unfortunately, none of the inhibitory anti-BamA mAbs bound to the K-12 strain by FACS, reconfirming that LPS likely masks the relevant or susceptible BamA epitope in the context of the K-12 strain. Despite this shortcoming, these inhibitory mAbs do validate the hypothesis that an anti-BamA antibody can result in direct growth inhibition of bacteria. This critical observation validates our key screening approach to use the Δ*waaD* strain of *E*. *coli* in order to maximize the potential to interrogate extracellular epitopes on BamA by mAbs. Moreover, these context-dependent mAbs represent valuable research tools to study BamA and BAM complex function by dissecting the mechanism of action of these rare growth inhibitory anti-BamA mAbs^30^.

## Discussion

Previous discovery efforts, including our own described above, have highlighted that solely relying on recombinant MPs as antigens can limit the success of an antibody discovery campaign. For example, different detergents or lipids can alter epitope conformations or global structures to generate proteins in non-native states^31,32^. Not surprisingly, relying solely on recombinant MP antigens to generate mAbs can lead to the discovery of detergent- and/or lipid-sensitive binders that fail to react with the native protein^17^. We observed that using whole bacteria to prime the animals followed by boosting with μg amounts of recombinant OMP protein enables robust generation of FACS^+^ pAbs and mAbs (Fig. 2). In total, we used only ~105 μg of recombinant MP antigen, 100 μg for boosting and 5 μg for sorting, to discover the first known anti-BamA mAbs with growth inhibitory activity. Our targeted boost-and-sort strategy is therefore particularly attractive for other dynamic OMP targets such as TonB-dependent receptors, autotransporters, or other virulence factors. Our method also alleviates the significant challenges and resources associated with generating high milligram amounts of recombinant material that are often required to support successful traditional mAb discovery workflows. It is therefore noteworthy that mAbs discovered from the targeted boost-and-sort method had comparably high-affinity (pM–nM range) and selectivity profiles to those mAbs that were discovered through more traditional, resource-intensive protein-based immunizations.

Whole cell-based immunizations are known to generate high levels of mAbs against non-target proteins, and thus, downstream mAb screening becomes extremely resource intensive. For the first time, to our knowledge, we established that sorting for Ag-specific hybridomas using amphipol-reconstituted MPs dramatically improves antibody discovery efforts by enriching the initial complex pool for antigen binding. Notably, sorting hybridomas with amphipol-reconstituted BamA greatly enriched the mAb pool for FACS binders (~53% of enriched IgG^+^ clones compared to 2.4% of non-enriched IgG^+^ clones), which also extended the epitope coverage across the target and likely enabled the identification of the rare bacteriostatic and bactericidal mAbs. We highlight the critical importance of having the target MP reconstituted in an appropriate matrix, since Ag-based sorting with detergent-solubilized BamA led to a loss of cell viability. Additionally, the amphipol matrix successfully masks the hydrophobic regions of BamA to help avoid the sorting of non-specific hybridomas. A variety of reconstitution methods are emerging to generate stabilized recombinant MPs which should greatly extend our methods to other MP targets; and in fact, we have already validated that several of these reconstitution formats also enable sorting of Ag-specific cells^12,16–19^.

We had hypothesized that an antibody might affect the function of BamA and result in bacterial growth inhibition by binding to an extracellular epitope and stabilize either an open, intermediate or closed conformation of BamA. Notably our own traditional protein antigen-only based mAb discovery approach as well as previous reports on BamA vaccine efforts had failed to identify such an antibody^33–35^. By contrast, using our new high-throughput targeted boost-and-sort platform, we rapidly identified BamA antagonist mAbs at a rate of 0.4% (7 inhibitors out of 1632 mAbs screened) that could completely inhibit growth of Δ*waaD E*. *coli*. We found that many non-inhibitory and partially inhibitory anti-BamA mAbs bin in the same epitope as the bactericidal mAb clones, suggesting small molecular differences in BamA binding can substantially impact antibody function. This key result suggests that broad coverage of a single epitope with a diverse panel of mAbs may be required to obtain functional mAbs against highly dynamic or especially challenging MP targets. While the present bactericidal mAbs were unable to bind their epitope in the presence of an intact LPS layer, future structural characterization of select mAbs may provide a starting point to engineer smaller scaffolds or peptides with the ability to penetrate the LPS layer and antagonize BamA^36^.

The conformational stabilization of mammalian MPs via mutagenesis or other methods can enable direct discovery of agonist or antagonist mAbs^12,37^. However, such stabilization methods typically require an existing tool compound to validate a given locked conformation, whereas for many MP targets (including BamA), such tools do not exist. Here, without such pre-existing tools, our efficient targeted boost-and-sort workflows robustly identified rare anti-BamA mAbs with bacterial growth inhibition activity, where our traditional methods failed to do so. One can therefore envision that our method may directly translate to more traditional targets like GPCRs; especially since numerous expression and stabilization methods to facilitate the production of ~100 μg levels of these high-value recombinant proteins have emerged in recent years^12,16–19^. Moreover, GPCRs and other mammalian targets are not masked by a surrounding LPS layer like BamA or other OMPs, so one might even anticipate a high discovery rate for binding antibodies (~50% FACS^+^) and rare modulators. Overall, we anticipate that the methods described herein will provide a resource-efficient approach for identifying novel mAb tools against additional MP targets to reveal new biological insights and enable the discovery or design of new therapeutics.

## Methods

### Protein expression and purification

The BamA gene lacking the N-terminal signal sequence and POTRA domains but encoding the beta-barrel outer membrane component from *Escherichia coli* (V418-W810) was cloned into a modified pET-52b expression vector containing and N-terminal His6 and Avi-tag. Protein was overexpressed using *E*. *coli* host BL21(DE3) grown by fermentation at 17 °C for 64 hours in autoinduction media. Cells expressing *E*. *coli* BamA were harvested and resuspended in buffer A (50 mM Tris pH 8.0, 500 mM NaCl, 0.1 mM TCEP), supplemented with cOmplete Protease Inhibitors (Roche), 1 mM PMSF and DNase. Following cell lysis by microfluidization, the insoluble BamA protein in inclusion bodies were harvested by low speed centrifugation (3,000 *g*) at 4 °C for 20 minutes and resuspended by brief vortexing in denaturing buffer B (50 mM Tris pH 8.0, 6 M GuHCl, 1 mM TCEP) followed by gentle agitation on a platform shaker for 1 hour at 22 °C. Insoluble debris and residual membrane fractions were pelleted by ultracentrifugation at >100,000 *g* at 4 °C for 1 hour using a Ti45 rotor. Supernatant was collected, protein concentration was determined (absorbance 280 nm), and the total protein concentration was adjusted to 1 mg/mL using denaturing buffer B. 40 mg of BamA was refolded by rapid dilution into a solution containing buffer C (1% (wt/v) zwittergent 3–12, 50 mM Tris pH 8.0, 0.1 mM TCEP) which was being mixed on a stir plate at 22 °C. The refolding reaction mixture was subsequently set at 22 °C without stirring for 2 days, then BamA was captured by batch binding using Ni-NTA resin. Resin was captured by gravity flow and washed with 5 column volumes of buffer D (1% (wt/v) zwittergent 3–12, 50 mM Tris pH 8.0) and eluted with 15 column volumes of the same buffer containing 300 mM imidazole pH 8.0. The eluted protein was concentrated in a centrifugal device (10 K MWKO) and applied over a Superdex 200 (26/60) column in buffer E (1.5% (wt/v) octyl-glucoside, 50 mM Tris pH 8, 100 mM NaCl). Protein in detergent for immunization was concentrated to 1 mg/mL using a centrifugal device (10 K MWKO; Millipore).

### Protein biotinylation, amphipol reconstitution and PE-labeling

When BamA protein was required for ELISA or antigen-based sorting, an *in vitro* biotinylation reaction using BirA enzyme targeting the N-terminal Avi tag was first carried out according to the manufacturers suggestions (Avidity); BamA was then rerun over the Superdex 200 (26/60) in buffer E as described above in order to remove free biotin and other reaction components. When protein was required in amphipol (either non-biotinylated or biotinylated), BamA at 1 mg/mL in buffer E was incubated with a stock solution of 2 mg/mL A8-35 amphipol (Anatrace) at 22 °C for 1 hour and then applied over a Superdex 200 (26/60) column in buffer F (50 mM Tris pH 8, 100 mM NaCl). Protein reconstituted in A8-35 amphipol for immunization was concentrated to 1 mg/mL using a centrifugal device (10 K MWKO; Millipore). When PE-labeled protein was required for antigen-specific sorting, BamA at 1 mg/mL (biotinyated and reconstituted in A8-35 amphipol in buffer F) was mixed 1:1 (vol/vol) with PE-streptavidin (Jackson ImmunoResearch) reconstituted in buffer F; the PE-streptavidin-biotin-BamA-amphipol complex was incubated at 22 °C for at least 15 minutes prior to use.

### Generation of murine hybridomas

All animal study designs for the mouse and rat immunizations were reviewed and approved by the Genentech Institutional Animal Care and Use Committee prior to the start of this work. All animal work was performed in accordance with relevant guidelines and regulations. Balb/c mice (Charles River Laboratories, Hollister, CA) were immunized with detergent-solubilized or amphipol-reconstituted *E*. *coli* BamA protein and either CFA or Ribi adjuvant (Sigma). pAbs were purified by Protein A and assayed by ELISA, FACS, and inhibition of bacterial growth as described below. Hybridoma fusions were performed as previously described and supernatants were screened for protein binding by ELISA^38^. All ELISA positive clones were purified and screened for inhibition of bacterial growth.

### Generation of rat hybridomas

Sprague Dawley rats (Charles River Laboratories, Hollister, CA) were immunized with *E*. *Coli* K12 cells in PBS (10^7^–10^9^ cfu via intravenous injection). The rats were then boosted four times with *E*. *Coli* K12 cells and a first set of sera was isolated (bleed #1). Protein A purified pAbs were then evaluated for binding by ELISA and inhibition of bacterial growth. Rats were further boosted twice with 10 μg *E*. *coli* BamA protein in amphipol and a second set of sera was isolated for analysis (bleed #2). To generate monoclonal antibodies, hybridoma fusions were performed as previously described except with a myeloma partner SP2ab that enables surface display of IgG cell^28,38^. After HAT selection in ClonaCell-HY Medium C (StemCell Technologies) for 4 days, hybridomas were stained with a cocktail of FITC-conjugated anti-rat IgG1/IgG2a/IgG2b mAbs (1:100 dilution; Bethyl Laboratories) and PE-conjugated BamA protein antigen (5 μg/μl). Samples were sorted on a FACSAria II cell sorter (BD Biosciences). Gating strategy to identify hybridoma cells was first based on size (FSC/SSC). After dead-cell exclusion with Propidium iodide (Sigma-Aldrich P-4864), IgG^**+**^BamA^**+**^ single cell hybridomas were sorted into 96-well tissue-culture plates containing 200 μl of ClonaCell-HY Medium E (StemCell Technologies). Profiles were analyzed by FlowJo v.9.7.7 software. Sorted cells were cultured for seven days.

### Hybridoma culture and purification

A semi-automated high throughput process for hybridoma culturing and antibody purification will be described elsewhere (Nakamura G., *et al*. manuscript in preparation). Briefly, individual hybridoma clones were transferred to 96-well tissue culture plates and cultured for 6–8 days. Cells were then transferred to 96-deep round well boxes and cultured for an additional 10 days in continuous orbital shaking incubators at 37 °C degrees. Supernatants were then harvested via centrifugation and IgG was purified from supernatants using MabSelect SuRe (GE Healthcare, Piscataway, NJ, USA). The eluted IgGs in 400 μL final volume were neutralized and normalized to 50–150 μg/mL prior to subsequent functional assay testing. IgG concentrations were determined by OD280 measurements.

### ELISA assays

Antibodies were screened by capture ELISA. Briefly, biotinylated detergent-solubilized BamA protein, diluted in assay buffer (PBS + 1.5% bOG + 0.5% BSA) was added to 384 well streptavidin-coated plates (Thermo Scientific USA) and incubated at 25 °C for 1 h. The plates were washed with wash buffer (PBS + 1.5% bOG). Hybridoma supernatants or purified antibodies were added to the wells and incubated at 25 °C for 1 h. The plates were washed with wash buffer. The captured antibody was detected with goat anti-rat HRP secondary antibody (Bethyl Laboratories USA). The plates were incubated at 25 °C for 1 h, washed with wash buffer and developed with TMB solution (Surmodics, USA). Plates were read at 630 nm.

### Mammalian flow cytometry assays

The variable light chain and variable heavy chain sequences of a murine anti-BamA hybridoma were determined using 5′ RACE followed by sequencing of the PCR amplified products. cDNAs encoded for both variable domains were gene synthesized (Genewiz) and cloned into a mammalian surface display vector. This vector contains the light chain connect to heavy chain via an IRES sequence and has a glycosylphosphatidylinositol linkage signal (GPI anchor) derived from human decay-accelerating factor fused to the C-terminus of the heavy chain to enable surface display of the IgG^29^. CHO cells that were transiently transfected with the cDNA encoding for the membrane-bound anti-BamA antibody were stained with streptavidin PE-conjugated biotinylated BamA protein antigen in 200 ul FACS staining buffer (PBS with 0.5% BSA and 2 mM EDTA) at a final concentration of 5 ug BamA/μl staining volume. Cells were stained for 15 min at 4 °C. After washing twice with PBS, cells were run on a BD LSRFortessa™ X-20 cell analyzer and data analyses was performed using FlowJo v.9.7.7 software.

### Bacterial flow cytometry assays

An *E*. *Coli* K12 glycerol stock was thawed in LB broth and incubated at 37 °C until reaching exponential phase of growth. Bacterial cells were then pelleted, re-suspended in ice-cold PBS with 1% BSA, and normalized to an OD600 of 0.5. Bacterial suspensions were diluted 1:2.5 in ice-cold PBS, 1% BSA and 2 × 10^6^ CFU were incubated with 10 µg/ml mAb or 40 µg/mL pAb at 4 °C for 1 h under gentle agitation. Bacterial cells were then washed three times in ice-cold PBS, 1% BSA and labeled using Alexa 488 anti-mouse IgG (H + L) (1:1000; Invitrogen) or Alexa 488 anti-rat IgG (H + L) (1:1000; Invitrogen) for 30 min 4 °C. Bacterial cells were then washed three times with ice-cold PBS, and fixed by adding 1 vol. of 2% w/v paraformaldehyde in PBS. Samples were run on a BD FACSCalibur and data were analyzed using FlowJo software.

### Bacterial growth assay

A kanamycin deletion-insertion mutation of *waaD* was obtained from the Keio collection^24^. The genotype of the parental BW25113 strain is: rrnB3 ΔlacZ4787 hsdR514 Δ(araBAD)567 Δ(rhaBAD)568 rph-1. The screening strain, BW25113 Δ*waaD* or parental BW25113, was grown to log phase in MHB II supplemented with 0.002% Tween-80 and 0.05% BSA, diluted to a final OD600 of 0.01 in sterile round-bottom 96-well plates. Antibodies were initially screened for activity at 10 μg/mL and growth inhibition was measured after 4 h of static incubation at 37 °C. A plate reader measured bacterial growth via optical density at 600 nm after shaking the plate for 25 s. For analysis of viable bacteria, colony-forming units (CFUs) were determined by plating cells on agar plates both prior to antibody addition and after 4 h of static incubation at 37 °C with 10 μg/mL antibody.

### Epitope binning and affinity characterization

Epitope bins were determined by 96 × 96 array-based SPR imaging system (Carterra, USA) using classical sandwich method. Purified antibodies were diluted at 20 μg/ml in 10 mM sodium acetate buffer pH 4.5. Using amine coupling, antibodies were directly immobilized onto a SPR sensorprism CMD 200 M chip (XanTec Bioanalytics, Germany) using a Continuous Flow Microspotter (Carterra, USA) to create an array of 96 antibodies. For antibody binning, printed chip was loaded on IBIS MX96 SPRi (Carterra USA) and *E*. *Coli* BamA protein, diluted to 50 nM in 1.5% bOG HBS-P buffer, was injected over the chip at 25 °C followed by a second injection of purified antibody, diluted at 20 μg/ml in 1.5% bOG HBS-P buffer, to make a sandwich. The epitope binning data was processed using Wasatch binning software tool.

### Statistics

All experiments involving bacterial growth were analyzed via the unpaired Student’s t test using Prism 7.0 (Graphpad Software) and compared mAb treated samples to the buffer controls.

## Electronic supplementary material

Supplementary Information

## References

[1] Wimley WC (2003). The versatile beta-barrel membrane protein. Curr Opin Struct Biol.

[2] Koebnik R, Locher KP, Van Gelder P (2000). Structure and function of bacterial outer membrane proteins: barrels in a nutshell. Mol Microbiol.

[3] Noinaj N, Gumbart JC, Buchanan SK (2017). The beta-barrel assembly machinery in motion. Nat Rev Microbiol.

[4] Malinverni JC (2006). YfiO stabilizes the YaeT complex and is essential for outer membrane protein assembly in Escherichia coli. Mol Microbiol.

[5] Gu Y (2016). Structural basis of outer membrane protein insertion by the BAM complex. Nature.

[6] Bakelar J, Buchanan SK, Noinaj N (2016). The structure of the beta-barrel assembly machinery complex. Science.

[7] Iadanza MG (2016). Lateral opening in the intact beta-barrel assembly machinery captured by cryo-EM. Nat Commun.

[8] Urfer M (2016). A Peptidomimetic Antibiotic Targets Outer Membrane Proteins and Disrupts Selectively the Outer Membrane in Escherichia coli. J Biol Chem.

[9] LaRocca TJ (2009). The bactericidal effect of a complement-independent antibody is osmolytic and specific to Borrelia. Proc Natl Acad Sci USA.

[10] Kruse AC (2013). Activation and allosteric modulation of a muscarinic acetylcholine receptor. Nature.

[11] Ring AM (2013). Adrenaline-activated structure of beta2-adrenoceptor stabilized by an engineered nanobody. Nature.

[12] Hutchings CJ (2014). Monoclonal anti-beta1-adrenergic receptor antibodies activate G protein signaling in the absence of beta-arrestin recruitment. MAbs.

[13] Oleksiewicz MB, Nagy G, Nagy E (2012). Anti-bacterial monoclonal antibodies: back to the future?. Arch Biochem Biophys.

[14] Pintor M (1996). Blocking of iron uptake by monoclonal antibodies specific for the Neisseria meningitidis transferrin-binding protein 2. J Med Microbiol.

[15] Huang JX, Bishop-Hurley SL, Cooper MA (2012). Development of anti-infectives using phage display: biological agents against bacteria, viruses, and parasites. Antimicrob Agents Chemother.

[16] Takeda H (2015). Production of monoclonal antibodies against GPCR using cell-free synthesized GPCR antigen and biotinylated liposome-based interaction assay. Sci Rep.

[17] Dominik PK (2016). Conformational Chaperones for Structural Studies of Membrane Proteins Using Antibody Phage Display with Nanodiscs. Structure.

[18] Tribet C, Audebert R, Popot JL (1996). Amphipols: polymers that keep membrane proteins soluble in aqueous solutions. Proc Natl Acad Sci USA.

[19] Knowles TJ (2009). Membrane proteins solubilized intact in lipid containing nanoparticles bounded by styrene maleic acid copolymer. J Am Chem Soc.

[20] Bond PJ, Sansom MS (2004). The simulation approach to bacterial outer membrane proteins. Mol Membr Biol.

[21] Patel DS, Qi Y, Im W (2017). Modeling and simulation of bacterial outer membranes and interactions with membrane proteins. Curr Opin Struct Biol.

[22] Bentley AT, Klebba PE (1988). Effect of lipopolysaccharide structure on reactivity of antiporin monoclonal antibodies with the bacterial cell surface. J Bacteriol.

[23] Pegues JC, Chen LS, Gordon AW, Ding L, Coleman WG (1990). Cloning, expression, and characterization of the Escherichia coli K-12 rfaD gene. J Bacteriol.

[24] Baba T (2006). Construction of Escherichia coli K-12 in-frame, single-gene knockout mutants: the Keio collection. Mol Syst Biol.

[25] Pocanschi CL, Popot JL, Kleinschmidt JH (2013). Folding and stability of outer membrane protein A (OmpA) from Escherichia coli in an amphipathic polymer, amphipol A8-35. Eur Biophys J.

[26] Li GW, Burkhardt D, Gross C, Weissman JS (2014). Quantifying absolute protein synthesis rates reveals principles underlying allocation of cellular resources. Cell.

[27] Masuda T, Saito N, Tomita M, Ishihama Y (2009). Unbiased quantitation of Escherichia coli membrane proteome using phase transfer surfactants. Mol Cell Proteomics.

[28] Price PW (2009). Engineered cell surface expression of membrane immunoglobulin as a means to identify monoclonal antibody-secreting hybridomas. J Immunol Methods.

[29] Akamatsu Y, Pakabunto K, Xu Z, Zhang Y, Tsurushita N (2007). Whole IgG surface display on mammalian cells: Application to isolation of neutralizing chicken monoclonal anti-IL-12 antibodies. J Immunol Methods.

[30] Storek KM (2018). Monoclonal antibody targeting the beta-barrel assembly machine of Escherichia coli is bactericidal. Proc Natl Acad Sci USA.

[31] Laganowsky A (2014). Membrane proteins bind lipids selectively to modulate their structure and function. Nature.

[32] Chung KY (2012). Role of detergents in conformational exchange of a G protein-coupled receptor. J Biol Chem.

[33] Guan Q, Wang X, Wang X, Teng D, Wang J (2016). In silico analysis and recombinant expression of BamA protein as a universal vaccine against Escherichia coli in mice. Appl Microbiol Biotechnol.

[34] Su YC, Wan KL, Mohamed R, Nathan S (2010). Immunization with the recombinant Burkholderia pseudomallei outer membrane protein Omp85 induces protective immunity in mice. Vaccine.

[35] Tashiro Y (2008). Opr86 is essential for viability and is a potential candidate for a protective antigen against biofilm formation by Pseudomonas aeruginosa. J Bacteriol.

[36] Kadam RU (2017). Potent peptidic fusion inhibitors of influenza virus. Science.

[37] Serrano-Vega MJ, Magnani F, Shibata Y, Tate CG (2008). Conformational thermostabilization of the beta1-adrenergic receptor in a detergent-resistant form. Proc Natl Acad Sci USA.

[38] Hazen M (2014). An improved and robust DNA immunization method to develop antibodies against extracellular loops of multi-transmembrane proteins. MAbs.

