# Supplementary material for: A targeted boost-and-sort immunization strategy using Escherichia coli BamA identifies rare growth inhibitory antibodies
Source: Sci Rep. 2018 May 8;8:7136. doi: 10.1038/s41598-018-25609-z (PMC5940829; doi:10.1038/s41598-018-25609-z)
Supplement: Supplementary file 1 — Supplementary Information [file 41598_2018_25609_MOESM1_ESM.pdf]

Title

A targeted boost-and-sort immunization strategy using *Escherichia coli* BamA identifies rare growth inhibitory antibodies

Authors

Rajesh Vij[1], Zhonghua Lin[1], Nancy Chiang[1], Jean-Michel Vernes[4], Kelly M. Storek[2], Summer Park[5], Joyce Chan[4], Gloria Meng[4], Laetitia Comps-Agrar[4], Peng Luan[1], Sophia Lee[1], Kellen Schneider[1], Jack Bevers III[1], Inna Zilberleyb[3], Christine Tam[3], Christopher M. Koth[3], Min Xu[5], Avinash Gill[1], Marcy R. Auerbach[2], Peter A. Smith[2], Steven T. Rutherford[2], Gerald Nakamura[1], Dhaya Seshasayee[1], Jian Payandeh[3*], James T. Koerber[1*]

[1]Department of Antibody Engineering, Genentech, 1 DNA Way, South San Francisco, California 94080, USA

[2]Department of Infectious Diseases, Genentech, 1 DNA Way, South San Francisco, California 94080, USA

[3]Department of Structural Biology, Genentech, 1 DNA Way, South San Francisco, California 94080, USA

[4]Department of Biochemical and Cellular Pharmacology, Genentech, 1 DNA Way, South San Francisco, California 94080, USA

[5]Department of Translational Immunology, Genentech, 1 DNA Way, South San Francisco, California 94080, USA

*Correspondence to J.P. (payandeh.jian@gene.com, 650-225-6416) and J.T.K. (koerber.james@gene.com, 650-467-4865)

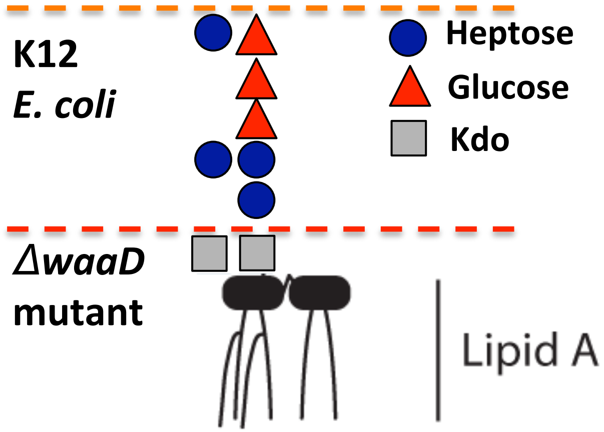

Supplemental Figure 1. Schematic of LPS structures on *waaD* and K12 strains. Diagram of LPS structure for both the K12 and waaD strains highlighting lack of elongated LPS in waaD (blue circle: heptose sugar; red triangle: glucose sugar; grey square: Kdo sugar).

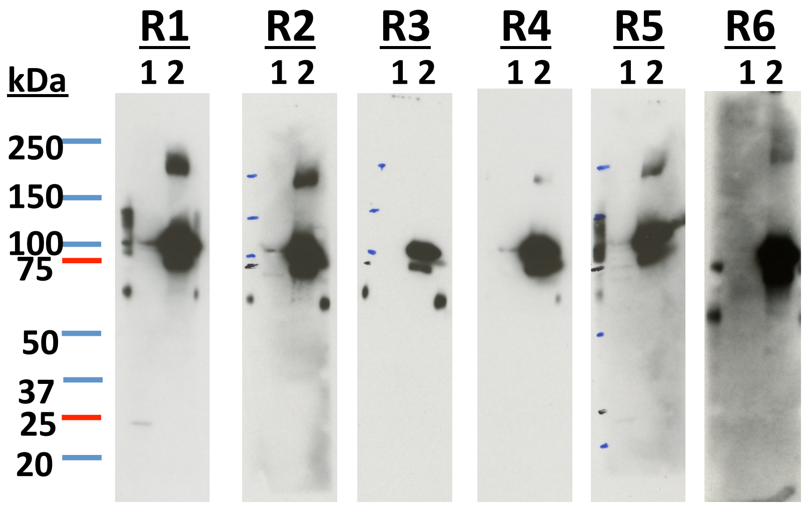

Supplemental Figure 2. Western blots against purified BamA with pAbs from K12 immunizations. Western blot results with 10 ug/mL of purified pAbs from bleed 1 are shown. Lane 1 contains 100 ng of an irrelevant protein and lane 2 contains 100 ng of recombinant BamA. All samples were run on a single gel and transferred to a blot. The blot was divided into five parts and each parts was stained with a different purified pAb sample. Images for the full blot are shown.

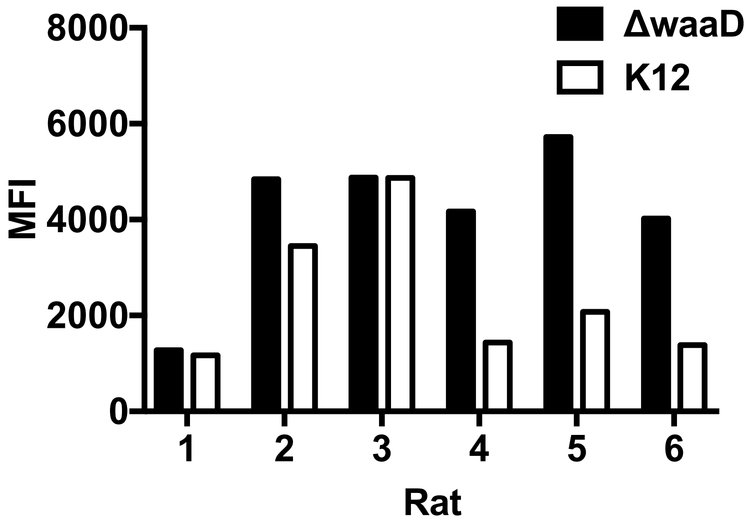

Supplementary Figure 3. MFI values for purified pAbs from whole cell immunization. MFI values on either *ΔwaaD* (black box) or K12 (white box) with 40 ug/mL purified pAbs for each of the six rats.

Format	Advantage	Disadvantage
Purified protein (detergent)	High purity	• Detergent-specific conformation<br>• Production
Purified protein (matrix)	• High purity<br>• Modular choice of lipids	• Lipid-specific conformation<br>• Production
Peptide	Ease of production	Hard to mimic native conformation
Membrane fraction	• Ease of production<br>• Native format	High level of non-target Abs
Membrane vesicle	• Ease of production<br>• Native format	• High level of non-target Abs<br>• Low target amount
Whole cell	• Ease of production<br>• Native format	• High level of non-target Abs<br>• Low target amount
DNA	• Ease of production<br>• Native format	• Low immune response<br>• Low expression level

Supplementary Table 1. Comparison of different antigen formats for membrane proteins. Overview of advantages and disadvantages in using different antigen formats for antibody discovery.
